# Evaluation of public health and economic impacts of dietary salt reduction initiatives on social security expenditures for cardiovascular disease control in Japan

**DOI:** 10.1038/s41440-025-02108-z

**Published:** 2025-02-17

**Authors:** Nayu Ikeda, Miwa Yamaguchi, Ikuko Kashino, Takehiro Sugiyama, Katsuyuki Miura, Nobuo Nishi

**Affiliations:** 1 National Institute of Health and Nutrition, National Institutes of Biomedical Innovation, Health and Nutrition, Osaka, Japan; 2 Diabetes and Metabolism Information Center, Research Institute, National Center for Global Health and Medicine, Tokyo, Japan; 3 Institute for Global Health Policy Research, Bureau of International Health Cooperation, National Center for Global Health and Medicine, Tokyo, Japan; 4 Department of Health Services Research, Institute of Medicine, University of Tsukuba, Ibaraki, Japan; 5 NCD Epidemiology Research Center, Shiga University of Medical Science, Shiga, Japan; 6 Graduate School of Public Health, St Luke’s International University, Tokyo, Japan

**Keywords:** Cardiovascular disease, Dietary salt reduction, Food environment, Simulation model, Social security costs

## Abstract

Japan has undertaken extensive efforts to reduce dietary salt intake and prevent cardiovascular diseases. Although salt consumption has decreased over time, levels remain high, highlighting the need for continued promotion of low-salt food products through collaboration among government bodies, the food industry, academia, and other stakeholders. Effective policy development requires an environment that enables stakeholders to apply scientific evidence on the cost-effectiveness of salt reduction strategies. Our ongoing research focuses on developing simulation models to predict future public health and economic impacts, supporting the establishment of voluntary targets and evidence-based approaches. These strategies aim to lower salt intake, enhance health outcomes, and manage social security expenditures, thereby fostering sustainable development in an aging society.

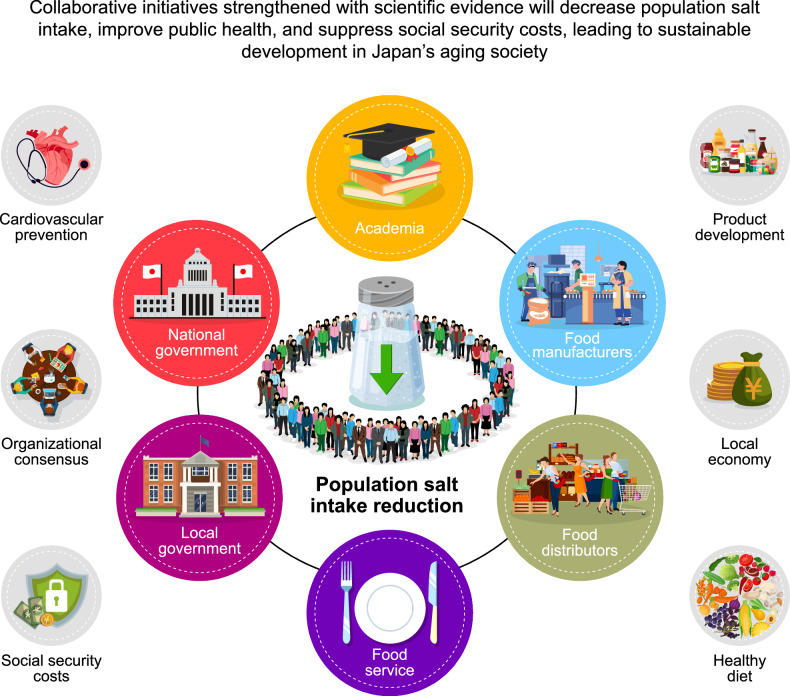

## Introduction

Japan faces a significant health policy challenge owing to rising social security expenditures associated with a rapidly aging population. In 2021, Japan had the highest life expectancy globally (84.5 years) and the second highest healthy life expectancy (73.4 years) [1]. Currently, 29% of the population is aged 65 or older [2], and this figure may rise to 36% by 2045 [3]. This demographic shift is placing increasing pressure on the public health system to manage the growing demand for healthcare, thereby threatening the long-term sustainability of the social security system. In 2021, national medical expenditures exceeded 8% of the gross domestic product, with over 60% allocated to older patients [4].

**Fig. 1:**
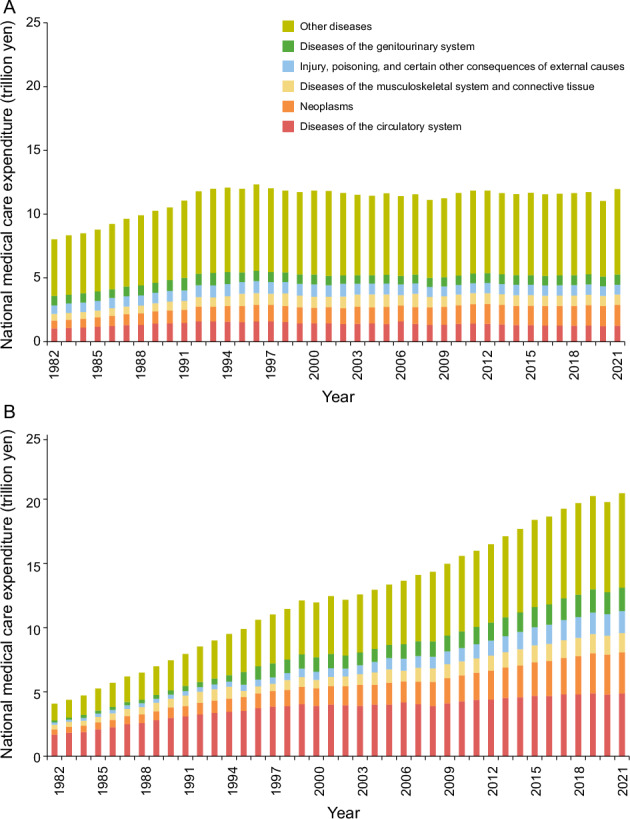
National medical care expenditure by disease in Japan, 1982–2021. **A** Patients under 65 years old; **B** Patients 65 years and older. Data are from the National Medical Expenditures [4]

Cardiovascular diseases (CVDs) are major contributors to national medical expenditures, accounting for 19% of total costs across all age groups and 24% among older patients (Fig. 1) [4]. CVDs have the highest prevalence among health conditions requiring treatment, with 20 million individuals seeking inpatient or outpatient care [5]. Managing CVDs necessitates extensive healthcare and nursing resources to continuously address the complications and disabilities that often arise after the acute phase, imposing a substantial long-term economic burden on society.

Excessive salt intake is the leading dietary risk factor for CVDs, contributing to 8% of deaths and disability-adjusted life years associated with these conditions in Japan [6]. Reducing dietary salt intake is a cost-effective strategy for controlling blood pressure and preventing CVDs [7–9]. Numerous simulation modeling studies have predicted the future health and economic impacts of salt reduction policies and interventions in various countries [10, 11]. This paper reviews the epidemiology of salt intake, the history of salt reduction initiatives, and related economic studies to establish a foundation for developing health and economic simulation models for salt reduction strategies in Japan.

## Trends in dietary salt intake

In 2019, the average daily salt intake for adults worldwide was 10.8 g [12]. National averages ranged from 5.0 g in Samoa to 17.4 g in China, with a median of 7.5 g in Namibia. No country met the target recommended by the World Health Organization (WHO) of less than 5 g [13]. Among the countries surveyed, twelve Central and Eastern European countries, including Hungary (14.1 g), followed China. Japan ranked 35^th^ highest with an intake of 10.1 g, whereas the Republic of Korea (12.1 g) and Singapore (11.3 g) ranked 15^th^ and 17^th^, respectively [12].

Japan has experienced a long-term reduction in dietary salt intake since the post-war era, as observed for example in a rural community in the northeast [14]. This decline may be partly attributed to the shift from traditional diets that relied on salt-preserved foods to Westernized eating patterns, driven by technological advancements and socioeconomic developments. Community-based nutrition education campaigns, particularly stroke prevention efforts, have also played a crucial role in reducing salt intake among the general population [15].

**Fig. 2:**
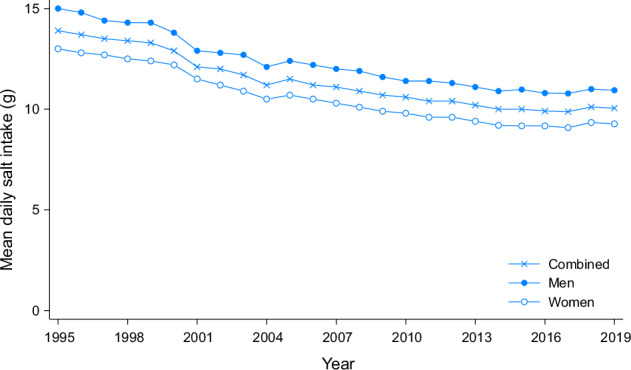
Mean dietary salt intake of adults aged 20 years and older in Japan by sex, 1995–2019. Data are from the National Nutrition Surveys (1995–2002) and the National Health and Nutrition Surveys (2003–2019) [16]

At the national level, the average daily salt intake among Japanese adults decreased from 13.9 g in 1995 to stabilize at just over 10 g by the mid-2010s (Fig. 2) [16]. However, substantial geographical variations exist across the country. In 2016, men in Miyagi, a northeastern prefecture, consumed an average of 11.9 g daily, compared with 9.1 g among men in Okinawa, the southernmost prefecture. Furthermore, the salt intake of women ranged from 8.0 g in Okinawa to 10.1 g in Nagano, located in the central part of the main island [17].

## Sources of dietary salt

**Fig. 3:**
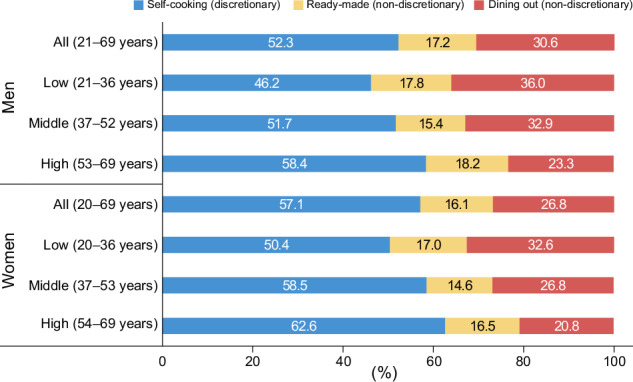
Proportions of dietary salt intake from self-cooking, ready-made foods, and dining out in Japanese adults by sex and age tertile in 2013. Data are from Asakura et al. (2016) [19]

Understanding the primary sources of dietary salt in a country is essential for developing effective salt reduction strategies. A systematic review of global dietary sources found an inverse correlation between gross domestic product per capita and the proportion of daily salt intake derived from salt added during cooking or at the dining table [18]. In high-income countries, these discretionary sources accounted for less than 25% of daily salt intake. However, in Japan, this proportion increased to over 50%, mirroring patterns observed in low- and middle-income countries such as Brazil and India [18]. A nationwide study conducted in 2013 revealed that the contribution of discretionary sources decreased among younger generations of Japanese adults (Fig. 3) [19]. In particular, men in their 20s to mid-30 s obtained more than half of their salt from processed foods and restaurant meals.

**Fig. 4:**
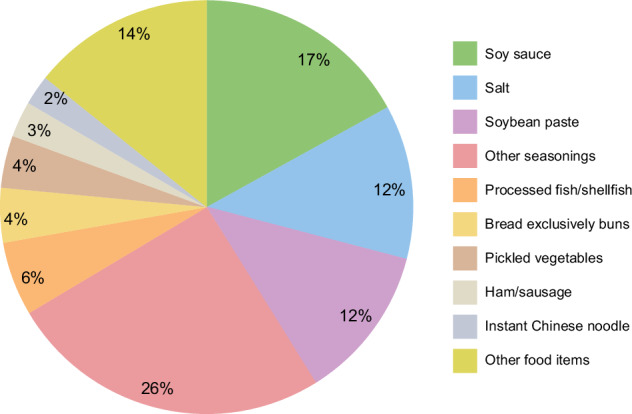
Sources of dietary salt intake among adults aged 20 years and older in Japan in 2019. Data are from the 2019 National Health and Nutrition Survey [21]

The systematic review further highlighted that, in most of the analyzed countries, the primary sources of dietary salt were processed foods such as bread, bakery products, cereals, grains, meat products, and dairy products [18]. However, 44% of the daily salt intake in Japan was attributed to sauces and dressings [18, 20]. According to the 2019 Japan National Health and Nutrition Survey, seasonings such as soy sauce and soybean paste accounted for 66% of the average daily salt intake, followed by processed fish and bread (Fig. 4) [21].

## Global salt reduction initiatives

The WHO has spearheaded global salt reduction efforts by providing comprehensive guidelines and resources for its member states (Table 1). Official documentation first emerged in the early 2000s when a joint WHO/Food and Agriculture Organization (FAO) expert consultation recommended maintaining daily salt intake below 5 g [22]. The initiative was further advanced in 2004 when the 57^th^ World Health Assembly endorsed the Global Strategy on Diet, Physical Activity, and Health, which promoted reducing salt consumption, ensuring salt iodization, and decreasing the salt content in processed foods [23].

**Table 1:** Key WHO salt reduction initiatives and publications

Year	Initiatives
2002	Joint WHO/FAO Expert Consultation on Diet, Nutrition and the Prevention of Chronic Diseases [22]
2004	Global Strategy on Diet, Physical Activity and Health [23]
2007	Prevention of Cardiovascular Disease: Guidelines for Assessment and Management of Cardiovascular Risk [24]
2010	WHO Package of Essential Noncommunicable (PEN) Disease Interventions for Primary Health Care [25]
2012	Guidelines on sodium intake [13]
2013	Global Action Plan for the Prevention and Control of Noncommunicable Diseases 2013–2020 [27]Implementation Tools: Package of Essential Noncommunicable (PEN) Disease Interventions for Primary Health Care in Low-Resource Settings [29]
2016	SHAKE the Salt Habit: the Shake Technical Package for Salt Reduction [31]
2017	Tackling NCDs: ‘best buys’ and other recommended interventions for the prevention and control of noncommunicable diseases [32]
2019	Global action plan for noncommunicable diseases expanded to 2030
2020	Package of Essential Noncommunicable (PEN) Disease Interventions for Primary Care [33]
2021	Global Benchmarks for Different Food Categories [34]
2023	Global Report on Sodium Intake Reduction [28]
2024	Tackling NCDs: Best Buys and Other Recommended Interventions for the Prevention and Control of Noncommunicable Diseases, second edition [36] Global Sodium Benchmarks for Different Food Categories, second edition [37]

*FAO* Food and Agriculture Organization, *NCDs* noncommunicable diseases, *WHO* World Health Organization

In 2007, the WHO introduced guidelines advising individuals to reduce their salt intake by at least one-third, aiming for less than 5 g per day [24]. This initiative was followed in 2010 by the WHO Package of Essential Noncommunicable (PEN) Disease Interventions for Primary Health Care [25]. In 2012, the WHO released additional sodium intake guidelines to support these targets, encouraging the food industry to lower salt levels in processed foods [13].

A pivotal development occurred in 2013 during the 66^th^ Session of the World Health Assembly when the WHO endorsed the Global Action Plan for the Prevention and Control of Noncommunicable Diseases (NCDs) for 2013–2020 [26, 27]. This plan introduced nine voluntary global targets, including a 30% relative reduction in average population salt intake by 2025, a target that is currently under review for extension to 2030 [28]. In addition, the PEN guidelines were updated to reinforce the recommendations to limit daily salt intake and reduce the consumption of processed foods [29].

Recognizing salt reduction as a cost-effective public health strategy, the WHO has aligned its efforts with Sustainable Development Goal 3.4, which aims to reduce premature mortality by one-third by 2030 [30]. To support nations in this mission, the WHO launched the SHAKE technical package in 2016, offering strategies for developing and monitoring salt reduction initiatives [31]. In 2017, the WHO identified four “Best-Buy” policies for salt reduction: mass media campaigns, public food procurement and service policies, reformulation targets for salt content in foods, and front-of-pack nutrition labeling [32].

In 2019, the global action plan for NCDs was extended to 2030, reaffirming the commitment of the WHO to salt reduction. The updated PEN guidelines released in 2020 emphasized the need to restrict salt intake to less than 5 g daily, reduce salt usage in cooking, and limit the consumption of processed and fast foods [33]. In 2021, the WHO established global sodium benchmarks for processed foods, encompassing 18 product categories [34].

In 2023, the WHO introduced a sodium country scorecard to evaluate national progress toward achieving the voluntary target of a 30% relative reduction in average population salt intake by 2025 [28]. The scorecard rates countries on a scale from 1 (lowest level) to 4 (highest level), based on the extent of implementation of salt reduction policies and other measures. As of March 2024, the scorecard indicated that eleven countries—Argentina, Brazil, Chile, Colombia, Czechia, Lithuania, Malaysia, Mexico, Saudi Arabia, Spain, and Uruguay—had implemented comprehensive packages, including at least two mandatory policies and all four “Best-Buy” interventions established by the WHO, achieving Level 4 [35]. Japan achieved Level 3 by enacting mandatory measures for sodium reduction, including food labeling standards and regulations for school lunches. In 2024, the WHO updated its intervention recommendations to include reformulation policies, mass media campaigns, protections against harmful food marketing, menu labeling, and portion size limitations [36]. The second edition of global sodium benchmarks, released the same year, expanded its coverage to 70 food subcategories [37].

## Japanese salt reduction initiatives

Japan has implemented various initiatives to reduce dietary salt intake and prevent CVDs (Table 2). In March 2000, three ministries—the Ministry of Education, Ministry of Health and Welfare, and Ministry of Agriculture, Forestry and Fisheries—introduced the Dietary Guidelines for Japanese, which recommended a daily salt intake of less than 10 g as part of a healthy eating strategy [38]. Concurrently, the Health Japan 21 initiative was launched in April 2000, aiming to decrease the average daily salt intake in adults from 13.5 g to 10 g by fiscal year 2010 [39].

**Table 2:** Salt reduction initiatives and targets in Japan

Year	Initiatives	Salt reduction target
2000	Dietary Guidelines for Japanese [38] Health Japan 21 (First Term) [39] JSH 2000 Guidelines [52]	10 g/day 10 g/day by 2010 7 g/day for hypertensive patients
2004	JSH 2004 Guidelines [53]	6 g/day for hypertensive patients
2005	2005 DRIs [40]	10 g/day (men), 8 g/day (women)
2009	JSH 2009 Guidelines [54]	6 g/day for hypertensive patients
2010	2010 DRIs [41]	9 g/day (men), 7.5 g/day (women)
2013	Health Japan 21 (Second Term) [45]	8 g/day by 2022
2014	JSH 2014 Guidelines [55]	6 g/day for hypertensive patients
2015	2015 DRIs [42]	8 g/day (men), 7 g/day (women)
2016	Revised Dietary Guidelines [44]	8 g/day (men), 7 g/day (women)
2019	JSH 2019 Guidelines [56]	6 g/day for hypertensive patients
2020	2020 DRIs [43]	7.5 g/day (men), 6.5 g/day (women)
2021	Tokyo Nutrition for Growth Summit [49]	
2022	Strategic Initiative for a Healthy and Sustainable Food Environment [50]	
2024	Health Japan 21 (Third Term) [46]	7 g/day by 2032

*DRIs* Dietary Reference Intakes, *JSH* Japanese Society of Hypertension

In April 2005, the Dietary Reference Intakes (DRIs) for Japanese adults established daily salt intake limits of less than 10 g for men and 8 g for women [40]. Subsequent revisions in 2010, 2015, and 2020 progressively lowered these targets to below 7.5 g for men and 6.5 g for women by 2020 [41–43]. In addition, the Dietary Guidelines were updated in June 2016 to recommend limits of under 8 g for men and 7 g for women [44].

To address ongoing public health challenges, the Health Japan 21 (Second Term) initiative was launched in 2013 with the goal of reducing average daily salt intake to 8 g by fiscal year 2022 [45]. However, by 2019, progress was limited: nearly 40% of adults consuming more than 8 g per day showed little interest in making dietary improvements [21]. In response, Health Japan 21 (Third Term) for 2024–2035 established a more ambitious goal of reducing average daily salt intake to 7 g by fiscal year 2032 [46].

Japan has also prioritized multisectoral collaboration to cultivate a healthy and sustainable food environment. From February to June 2021, the Ministry of Health, Labor, and Welfare (MHLW) convened an expert review committee comprising relevant ministries and agencies to discuss strategies for fostering an environment that promotes healthier food choices [47]. The committee identified three priority issues: excessive salt intake, underweight among young women, and nutritional disparities. Acknowledging the role of businesses in environmental sustainability, the committee advocated for collaboration to develop and promote nutritionally and environmentally conscious food products.

Japan committed to a comprehensive policy package addressing the three key priorities during the Tokyo Nutrition for Growth (N4G) Summit in December 2021 [48]. These commitments were formalized in the Tokyo Nutrition Declaration (Tokyo Compact on Global Nutrition for Growth) [49], which consolidated pledges from various stakeholders, including the national government and major global institutional investors. The declaration focused on advancing nutrition policies, promoting a healthy and sustainable dietary environment, and creating a multisectoral collaboration mechanism for nutrition improvement.

Building on the committee report and the commitments made at the N4G Summit, and in collaboration with the Consumer Affairs Agency and Ministry of the Environment, the MHLW launched the Strategic Initiative for a Healthy and Sustainable Food Environment in March 2022 [50]. This initiative aims to address nutritional and environmental challenges by creating a food environment where healthier choices are the default. It promotes the reformulation of food products to reduce salt content and encourages businesses to establish and promote voluntary targets. The WHO has identified this initiative as one of 95 multisectoral actions across 46 countries, regions, and territories [51]. The Japanese initiative was the only one focusing on unhealthy diets in high-income countries and was selected as one of 20 for further exploration in case studies.

## Initiatives of the Japanese Society of Hypertension (JSH)

In its 2000 guidelines, the JSH initially recommended that patients with hypertension limit daily salt intake to less than 7 g (Table 2) [52]. As evidence of the health risks associated with excessive salt consumption accumulated, the JSH revised its guidelines in 2004, lowering the target to less than 6 g [53]. Subsequent updates published in 2009, 2014, and 2019 have maintained this recommendation [54–56].

Since 2013, the Salt Reduction Committee of the JSH has actively promoted low-salt food products by maintaining a list of approved items to encourage food manufacturers. As of April 2024, this list included 108 products from 26 companies [57]. The JSH conducts annual surveys on the sales of these products, which revealed that the salt content in 112 products from 26 companies decreased by 1148 tons in fiscal year 2023, with 79% of this reduction attributable to seasonings and the remaining portion to processed foods [58]. Since 2013, the cumulative salt reduction across 292 products from 41 companies has reached 9678 tons.

## Basic research on the impact of salt reduction policies

Evaluating the cost-effectiveness of nutritional policies is essential for managing rising social security expenditures. Three authors (N.I., T.S., and principal investigator N.N.) conducted basic health economics research focused on controlling social security costs through nutritional policies [59]. Funded by the MHLW from 2019 to 2021, this research aimed to develop methods for assessing how nutritional policies, including salt reduction initiatives, could curb the increase in social security costs by preventing diseases and disabilities. The study drew on findings from similar research conducted internationally.

A literature review was conducted to evaluate health economic studies on population-wide dietary salt reduction policies for preventing CVDs in various countries [60]. The review aimed to guide the development of methods to assess the effects of nutritional policies on public health and social security costs in Japan. Key health economic simulation models identified include the Cardiovascular Disease Policy Model [61, 62], IMPACT Coronary Heart Disease Policy and Prevention Model [63–65], US IMPACT Food Policy Model [66, 67], Assessing Cost-Effectiveness approach to priority-setting [68], and Prevention Impacts Simulation Model (PRISM) [69, 70]. These models employ techniques such as Markov cohort simulations, microsimulations, proportional multistate life tables, and system dynamics. They have been applied in countries such as Australia, England, and the United States to evaluate salt reduction strategies, including health promotion campaigns, sodium labeling, and food industry reformulation.

Following the literature review, three simulation studies were conducted to assess the impact of salt reduction policies on CVD prevention in Japan, using existing data from published studies and official statistics [71–73]. The first study developed and employed a system dynamics model to retrospectively estimate the reduction in cardiovascular deaths associated with decreased average salt intake from 1950 to 2017 [71]. The simulation of a counterfactual scenario, where salt intake remained unchanged, indicated that the observed decline in salt intake since the 1950s prevented approximately 298,000 deaths in men and 118,000 in women. The second study used a Markov model to project the effects of achieving salt reduction targets set by Health Japan 21 (Second Term), the JSH 2000 guidelines, and the WHO on cardiovascular events and healthcare spending [72]. The model estimated that meeting these targets over a ten-year period from 2019 could prevent 1–3% of cardiovascular events and reduce related healthcare costs by up to 2%, reflecting moderate health economic benefits. Furthermore, the third study examined hypothetical scenarios of implementing salt reduction policies from England in Japan, including media health promotion campaigns, front-of-pack labeling, voluntary reformulation, and mandatory reformulation [73]. A Markov cohort simulation model was constructed based on published data on these policies from England [63], as equivalent data for Japan were unavailable. The simulation showed that voluntary and mandatory reformulation without policy costs generated the greatest net benefits over a ten-year period.

## Research on the impact of salt reduction in food environment initiatives

Access to scientific evidence is essential for businesses and local governments involved in the Healthy and Sustainable Food Environment Strategy Initiative. To meet this need, the authors initiated research funded by the MHLW in 2023 (principal investigator: N.I.) [74]. Building on previous foundational research, this study aims to predict the future health and economic effects of salt reduction policies and interventions, including voluntary reformulation of food products. In addition, it seeks to provide practical resources, such as guidelines and simulation platforms, to support informed decision-making.

The research began with a comprehensive review of salt reduction goals and initiatives voluntarily adopted by food manufacturers across various countries. A questionnaire survey was conducted to examine corporate practices related to target setting and salt reduction efforts, particularly in countries where governments recommend voluntary actions. Government guidelines for food companies undertaking voluntary reformulation were also reviewed, with a focus on Canada, the United Kingdom, and the United States. Based on these findings, a support guide is being developed to assist domestic food-related businesses in setting voluntary salt reduction targets that align with national dietary goals and WHO guidelines.

The research will also develop a simulation model to estimate the public health and economic impact of salt reduction strategies across each prefecture. To support this endeavor, a literature review was conducted on existing studies that simulate the effects of salt reduction at subnational levels in other countries. The review highlighted the PRISM system dynamics simulation model [75], a web platform developed by the U.S. Centers for Disease Control and Prevention to support local authorities. Using PRISM as a reference, a simulation platform will be created for collaborative use, along with a guide to assist local governments in applying these models for evidence-based policymaking on salt reduction. These outputs will provide robust scientific evidence to support the development of food environments that promote lower salt intake.

## Conclusion

Despite continuous initiatives, salt intake in Japan remains high compared with that in many other nations. Achieving salt reduction targets calls for intensified efforts to reformulate food products and lower salt content through a collaborative approach involving the government, food industry, academia, and other stakeholders. Reducing population salt intake is a cost-effective approach to preventing CVDs. Creating an environment where stakeholders can apply scientific evidence on the health and economic impacts of salt reduction is essential for informed policymaking. Ongoing research is progressing in this direction, aiming to deliver robust evidence and practical tools to support these efforts. Strengthening these initiatives and implementing evidence-based strategies will be vital for achieving substantial reductions in population salt intake, enhancing public health outcomes, and managing rising social security costs, thereby contributing to sustainable societal development.
